# Matrix-assisted laser desorption/ionization mass spectrometry imaging (MALDI-MSI) reveals potential lipid markers between infrapatellar fat pad biopsies of osteoarthritis and cartilage defect patients

**DOI:** 10.1007/s00216-023-04871-9

**Published:** 2023-07-28

**Authors:** Mirella J. J. Haartmans, Britt S. R. Claes, Gert B. Eijkel, Kaj S. Emanuel, Gabrielle J. M. Tuijthof, Ron M. A. Heeren, Pieter J. Emans, Berta Cillero-Pastor

**Affiliations:** 1 Division of Imaging Mass Spectrometry, Maastricht MultiModal Molecular Imaging Institute (M4i), Maastricht University, Maastricht, the Netherlands; 2 Laboratory for Experimental Orthopedics, Department of Orthopedic Surgery, Joint Preserving Clinic, CAPHRI Care and Public Health Research Institute, Maastricht University Medical Center+, Maastricht, the Netherlands; 3 Department of Orthopedic Surgery and Sport Medicine, Amsterdam Movement Sciences, Amsterdam UMC, Amsterdam, the Netherlands; 4 Biomedical Device Design and Production Technology, Faculty of Engineering Technology, University of Twente, Enschede, the Netherlands; 5 MERLN Institute for Technology-Inspired Regenerative Medicine, Department of Cell Biology-Inspired Tissue Engineering (cBITE), Maastricht University, Universiteitssingel 40, 6229 ER Maastricht, the Netherlands

**Keywords:** Hoffa’s fat pad, Cartilage regeneration, Total knee arthroplasty, Mass spectrometry imaging, Biomarkers, Lipids

## Abstract

The incidence of osteoarthritis (OA) has been expected to increase due to an aging population, as well as an increased incidence of intra-articular (osteo-) chondral damage. Lipids have already been shown to be involved in the inflammatory process of OA. This study aims at revealing region-specific lipid profiles of the infrapatellar fat pad (IPFP) of OA or cartilage defect patients by matrix-assisted laser desorption/ionization mass spectrometry imaging (MALDI-MSI), which could be used as biomarkers for early OA detection. A higher presence of phospholipids was found in OA patients compared with cartilage defect patients. In addition, a higher abundance of ether-linked phosphatidylethanolamines (PE O-s) containing arachidonic acid was specifically found in OA patients compared with cartilage defect patients. These lipids were mainly found in the connective tissue of the IPFP. Specific lipid species were associated to OA patients compared with cartilage defect patients. PE O-s have been suggested as possible biomarkers for OA. As these were found more abundantly in the connective tissue, the IPFP’s intra-tissue heterogeneity might play an important role in biomarker discovery, implying that the amount of fibrous tissue is associated with OA.

**Graphical Abstract:**

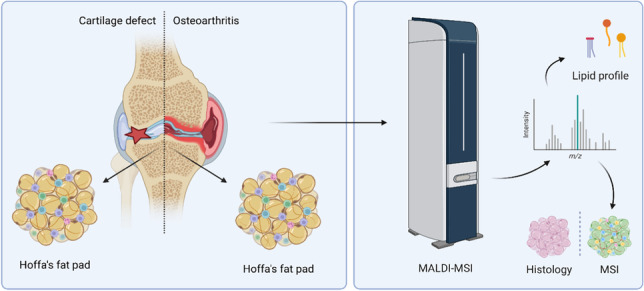

**Supplementary Information:**

The online version contains supplementary material available at 10.1007/s00216-023-04871-9.

## Introduction

Knee osteoarthritis (OA) is one of the leading causes of disability in the elderly population due to its high prevalence [1]. While OA is affecting millions of patients already, its incidence is expected to increase over the next years due to our aging population [2, 3]. OA development early in life is often related to an increased incidence of diabetes mellitus type 2 and obesity [2, 3], but also to an increase in intra-articular or osteochondral damage caused by (sport) injuries or trauma [4, 5]. As a result, total knee replacement surgery is being conducted at a younger age, resulting in higher chances of prosthesis revision [6] and immobility later in life. Considering this, the urge for joint-preserving or preventive therapy has never been stronger.

Cartilage has a limited ability to regenerate due to a lack of vasculature and a low abundance of cells [7]. When treated insufficiently, cartilage damage may lead to the development of OA [8]. This could be the result of changes in the cartilage matrix structure, leading to degradation of matrix or collagen type II [9, 10]. Additionally, accompanying inflammation, the release of pro-inflammatory cytokines [9, 11] accelerates cartilage degradation, resulting in OA development.

Currently used (surgical) treatments after a cartilage defect, to prevent OA from developing, focus on regeneration (minced cartilage, autologous chondrocyte transplantation, or hedgehog) or resurfacing (autograft or allograft transplantation and metal implant) [12–15]. Less common, but not less important, are treatments like osteotomy, joint distraction, or pharmaceuticals to prevent further OA development [16–18]. The major hurdle for successful treatment is the early detection of cartilage damage and finding the right treatment for the right patient. Therefore, biomarker research is needed to fill this gap [19]. These biomarkers can be derived invasively from tissues or fluids inside the knee (proteins, peptides, metabolites, lipids, genes) but also non-invasively from follow-up imaging (magnetic resonance imaging (MRI) or radiographic imaging) or questionnaires [20]. A clear predictive biomarker, indicating if and which joint-preserving treatments might have an OA disease-modifying effect, could further facilitate the clinical decision-making of these joint-preserving treatments and improve patient outcomes.

OA has been acknowledged as a whole-joint disease, affecting not only cartilage, but also other intra-articular tissues such as the meniscus, synovial membrane, or infrapatellar fat pad (IPFP) [21–23]. The IPFP, or Hoffa’s fat pad, is a piece of fat tissue located underneath the patella (tendon) [23, 24] and is often removed (partially) to gain access to the knee capsule during surgery. It is already known to be involved in the inflammatory process of OA by secretion of pro-inflammatory factors such as prostaglandins [23, 25] and might therefore be a potential source for biomarker discovery.

Since IPFP can be used as an accessible biopsy target, molecular markers found in the IPFP may have potential as diagnostic biomarkers in the use of joint-preserving treatments and the development of (early) OA. Histologically, the IPFP is a heterogeneous tissue, containing adipose, fibrous, and synovial tissue, as well as blood vessels. Due to its heterogeneous composition, matrix-assisted laser desorption/ionization mass spectrometry imaging (MALDI-MSI), a technique to study the spatial distribution of molecules and possible detection of biomarkers, could reveal local tissue-specific molecular changes [26].

MALDI-MSI has already been used in the past in the field of biomedical research [27], enabling the visualization of the spatial distribution of molecules in sections of tissues, to identify possible diagnostic compounds or biomarkers [28–30]. The analysis of lipids is already a growing area in the MSI field [31–33]. OA in the knee has been associated with an increased inflammatory profile and increased secretion of pro-inflammatory factors by the IPFP [23, 34]. Lipids have been shown to play an important role in OA [35] in which lipid deposits are stored in chondrocytes of OA articular cartilage [35, 36] and elevated levels of fatty acids have been associated with the histological severity of OA [35]. Additionally, it has been hypothesized that an altered lipid metabolism could lead to a variety of changes related to OA [37].

This study aims to identify the differences in lipid profiles of the IPFP between OA and cartilage defect patients; and to identify OA and cartilage defect-specific lipids which could be used as a biomarker for OA development. In addition, the influence of the two main tissue types in the IPFP (adipose tissue and connective tissue) on the lipid profile, and its effect on characterizing OA and cartilage defect patients, was investigated.

## Materials and methods

### Patient inclusion and tissue collection

Human IPFP was collected as surgical waste material from seven patients with moderate- to late-stage OA undergoing total knee arthroplasty (TKA), and seven control patients undergoing cartilage repair surgery for an osteochondral defect in a femoral condyle. Patients were matched for age (median 50.4 (OA) and 43.9 (cartilage defect), ns), gender (4 OA females, 3 cartilage defect females, ns), and body mass index (BMI, median 27.3 (OA) and 27.1 (cartilage defect), ns) regarding the availability of tissue. The Medical Ethics Committee (MEC) assigned non-WMO (wet medisch-wetenschappelijk onderzoek, law for medical-scientific research in humans in the Netherlands) approval for the collection of waste material during TKA (ID: MEC 2017–0183, 2017) or cartilage repair surgery (ID: MEC 2018–0963, 2018). In addition, patients signed informed consent for the use of this waste material for experimental purposes. Patients were graded for radiographic OA severity using the Kellgren-Lawrence (KL) score (3.3 for OA vs 0.3 for cartilage defect, *p* < 0.001) by an orthopedic surgeon (PE).

### Sample preparation and mass spectrometry imaging

The sample preparation workflow has been previously described in detail [26]. In summary, directly after dissection, every IPFP was washed in phosphate-buffered saline (PBS), snap-frozen in liquid nitrogen, and stored at – 80 °C until further processing. Frozen IPFPs were sectioned at 15-μm thickness with a cryostat (Leica Microsystems Cryotome, Wetzlar, Germany) in the presence of dry ice (cryotome temperature below – 30 °C) and mounted onto indium tin oxide (ITO)–coated glass slides (Delta Technologies, CO, USA). All tissue sections were sublimed (HTX Sublimator, HTX Imaging, Chapel Hill, NC, USA) with 55 ± 2 mg Norharmane matrix (Sigma-Aldrich, Zwijndrecht, the Netherlands) to extract lipids (preheating at 60 °C, pressure at < 0.04 mBar, sublimation at 140 °C for 200 s). Lipid analysis was performed by MALDI-MSI in positive and negative ion modes on consecutive sections at a lateral resolution of 50 μm on a RapifleX Tissue Typer (Bruker Daltonics, Bremen, Germany), running in reflectron mode. The instrument was calibrated using a standard mixture of red phosphorous (Sigma-Aldrich, Saint Louis, MO, USA). Lipids were detected over a mass range of *m/z* (mass-to-charge ratio) 100–2000 Da.

### Lipid identification

The identification of lipids was performed on an Orbitrap-Elite mass spectrometer (Thermo Fisher Scientific, Waltham, MA, USA) running in data-dependent acquisition (DDA, positive and negative) mode [26, 38], on consecutive sections at a spatial resolution of 100 µm and a laser repetition rate of 1000 Hz. Here, mass spectrometry (MS)^1^ data of *m/z* 200–2000 were acquired at a nominal mass resolution of 240,000 (FWHM *m/z* 400) with an injection time of 250 ms. In parallel, MS^2^ data were acquired in the ion trap with collision-induced dissociation (CID) using an isolation window of 0.7 Da. A normalized collision energy (NCE) of 30 (manufacturer units) and an activation *Q* of 0.17 were used in positive mode, and 38 and 0.25 in negative mode, respectively. A selected number of lipids with inclusive spectra were manually fragmented in the higher-energy collisional dissociation (HCD) cell using an injection time of 2000 ms and an NCE between 25 and 30 for 30 scans, while continuously moving the stage.

### Histology

To correlate the tissue structure to the MSI data, the same tissue sections were stained with Mayer’s Hematoxylin (Dako, Agilent Technologies, Glostrup, Denmark) and Eosin (Merck KGaA, Darmstadt, Germany) (H&E) after MALDI-MSI using a standard protocol to correlate the IPFP’s tissue structure to the MSI data. In brief, the matrix was removed from the slide by incubating the slide in 70% ethanol for 5 min. Subsequently, slides were immersed in distilled water for 3 min. Then, the tissue sections were stained with Mayer’s Hematoxylin (Dako, Agilent Technologies, Glostrup, Denmark) for 3 min before being rinsed under running tap water for 3 min. After the slides were rinsed with distilled water for 30 s, tissue sections were stained with Eosin (Merck KGaA, Darmstadt, Germany) for 30 s. To remove an excess of eosin, the slides were dipped 10 times in 70% ethanol. Subsequently, the slides were dehydrated in 100% for 5 min twice. Then, the slides equilibrated in xylene for 5 min twice. The sections were mounted with Histomount (Sigma-Aldrich) and covered with a glass coverslip. Microscopic images were taken with an Aperio CS2 with a 20 × objective (Leica Biosystems, Wetzlar, Germany).

### Data analysis

Using a Kolmogorov–Smirnov normality test with Dallal-Wilkinson-Lillie for *p* value (*p* < 0.05), normal distribution was tested. Differences between groups regarding age, gender, BMI, left or right knee, and KL score were determined using an unpaired nonparametric Mann–Whitney test in GraphPad Prism version 8.1.2 (GraphPad Software, San Diego, CA, USA) to compare ranks (*p* < 0.05).

All files were exported from FlexImaging 5.0 (MS^1^, Bruker Daltonics, Bremen, Germany) as imzML and loaded in SCiLS lab 2019b (SCiLS lab, GmbH, Bremen, Germany) software. From here, data were exported to MATLAB 2016b (The MathWorks, Natick, MA, USA). A region of interest (ROI), including the tissue types of interest (adipose tissue or connective tissue), based on histological examination, was selected for each tissue section using in-house ChemomeTricks data analysis tool for MATLAB.

Additionally, tissue sections were assigned to either OA or cartilage defect groups for spectrum-based principal component analysis (PCA), followed by linear discriminant analysis (LDA) to analyze the differences between patient groups and tissue types (Supplementary Fig. 1). Score projections were performed in the same program. A region of interest, based on the H&E images of both adipose tissue and connective tissue, was selected for further analysis. Lipid species were manually assigned using MS^1^ and MS^2^ spectra acquired from DDA measurements using Freestyle software (Thermo Fischer Scientific), Lipostar MSI version 1.1.0b26 (MS^2^, Molecular Discovery, Borehamwood, UK) using the LIPID MAPS database (3- and 4-star rating, Molecular Horizon, Bettona, PG, Italy) [39] and the ALEX123 web application [40]. The top 20 lipids showing the highest loading for each group (OA, cartilage defect, adipose tissue, or connective tissue) in negative and positive ion mode after PCA and LDA were selected for identification.

## Results

### Patient characteristics

In this study, lipid profiles in the IPFP of patients with late-stage OA were compared with those of patients undergoing cartilage repair surgery for a cartilage defect. Patient characteristics, including age, BMI, gender, and KL score, are depicted in Table 1. In addition, found lipid species were identified and IPFP intra-tissue heterogeneity was evaluated. An example of IPFP intra-tissue heterogeneity is visualized in Supplementary Fig. 2.

**Table 1:** Characteristics of osteoarthritis (*n* = 7) and cartilage defect (*n* = 7) patients undergoing total knee arthroplasty or cartilage repair surgery

Patient	OA/CD	Gender	Age	BMI	KL grade	Female (*n*)	Age (med.)	BMI (med.)	KL (med.)
1	OA	Male	56	26.9	3	4	50.4	27.3	3.3
2	OA	Male	42	29.6	3				
3	OA	Female	60	25.7	4				
4	OA	Female	54	28.3	4				
5	OA	Male	52	23.2	4				
6	OA	Female	58	26.0	3				
7	OA	Female	40	31.5	2				
8	CD	Male	47	23.7	1	3 (ns)	43.9 (ns)	27.1 (ns)	0.3^***^
9	CD	Male	38	34.3	0				
10	CD	Female	49	24.3	0				
11	CD	Male	48	26.6	0				
12	CD	Male	33	31.0	0				
13	CD	Female	56	27.2	0				
14	CD	Female	36	22.5	1				

*OA* osteoarthritis, *CD* cartilage defect, *BMI* body mass index, *KL* Kellgren-Lawrence, *ns* not significant, *med*. median

^***^p < 0.001

The data regarding patient characteristics did not show a normal distribution. Gender, age, and BMI were not statistically different between groups (*p* > 0.05). The KL score was significantly decreased in the cartilage defect group compared to the OA group (*p* < 0.001).

### MALDI-MSI approach on the IPFP

As described before [26], the main challenge is the IPFP’s adipose-like structure. Sample handling and sample preparation are of importance while applying MALDI-MSI on the IPFP to prevent delocalization of molecules. The most important aspects of the working method included cryosectioning at 15 µm below a temperature of – 30 °C, the use of an anti-roll system, quick thaw mounting and refreezing, and matrix application by sublimation. Furthermore, it has been shown of importance that the slides were transported inside a silica carrier box and dried thoroughly in a desiccator before matrix application.

### Patient profiling based on IPFP lipid composition in negative ion mode

**Fig. 1:**
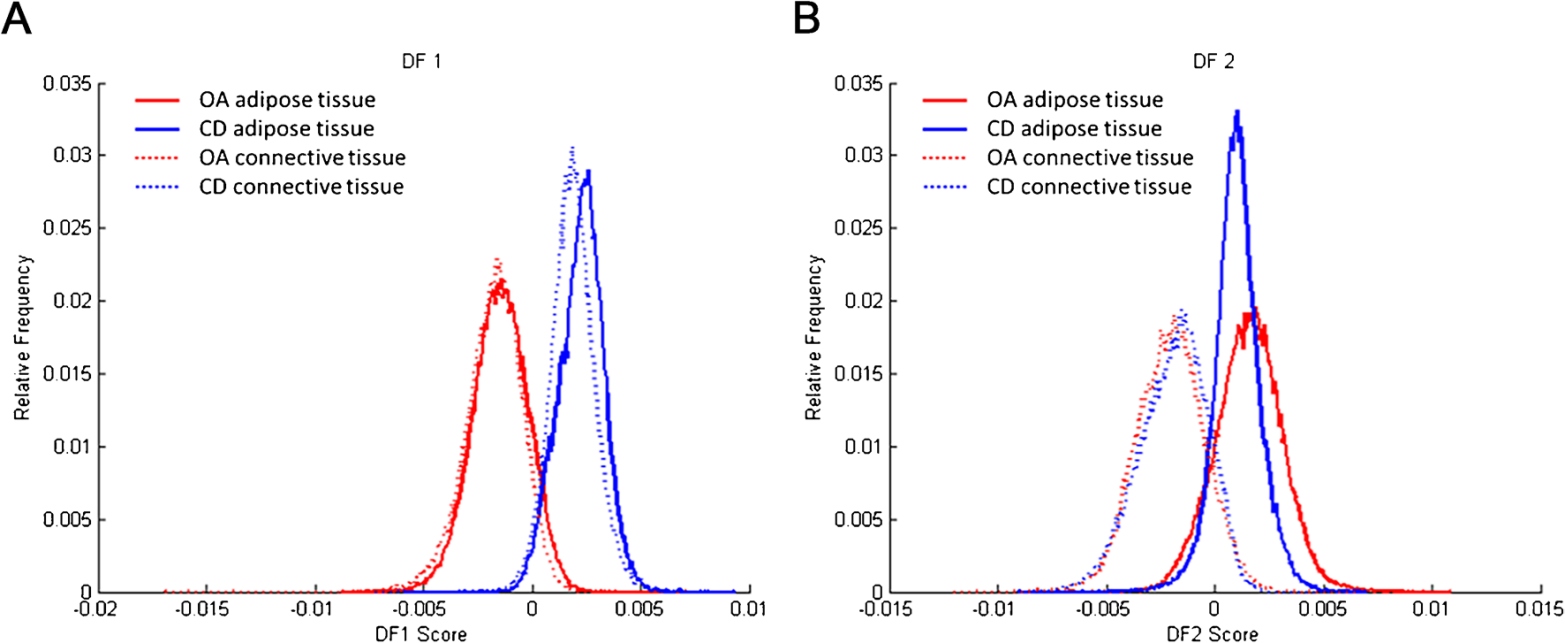
Discriminant function 1 (DF1) discriminates between osteoarthritis (OA) and cartilage defect (CD) patients, independently from tissue type (adipose tissue or connective tissue) after principal component analysis (PCA) and discriminant analysis (DA) in negative ion mode (**A**, between/within (B/W) = 2.4). Additionally, DF2 showed discrimination between adipose tissue (AT) of OA and CD patients and connective tissue (CT), independently from patients’ disease pathology (**B**, B/W = 1.3)

**Fig. 2:**
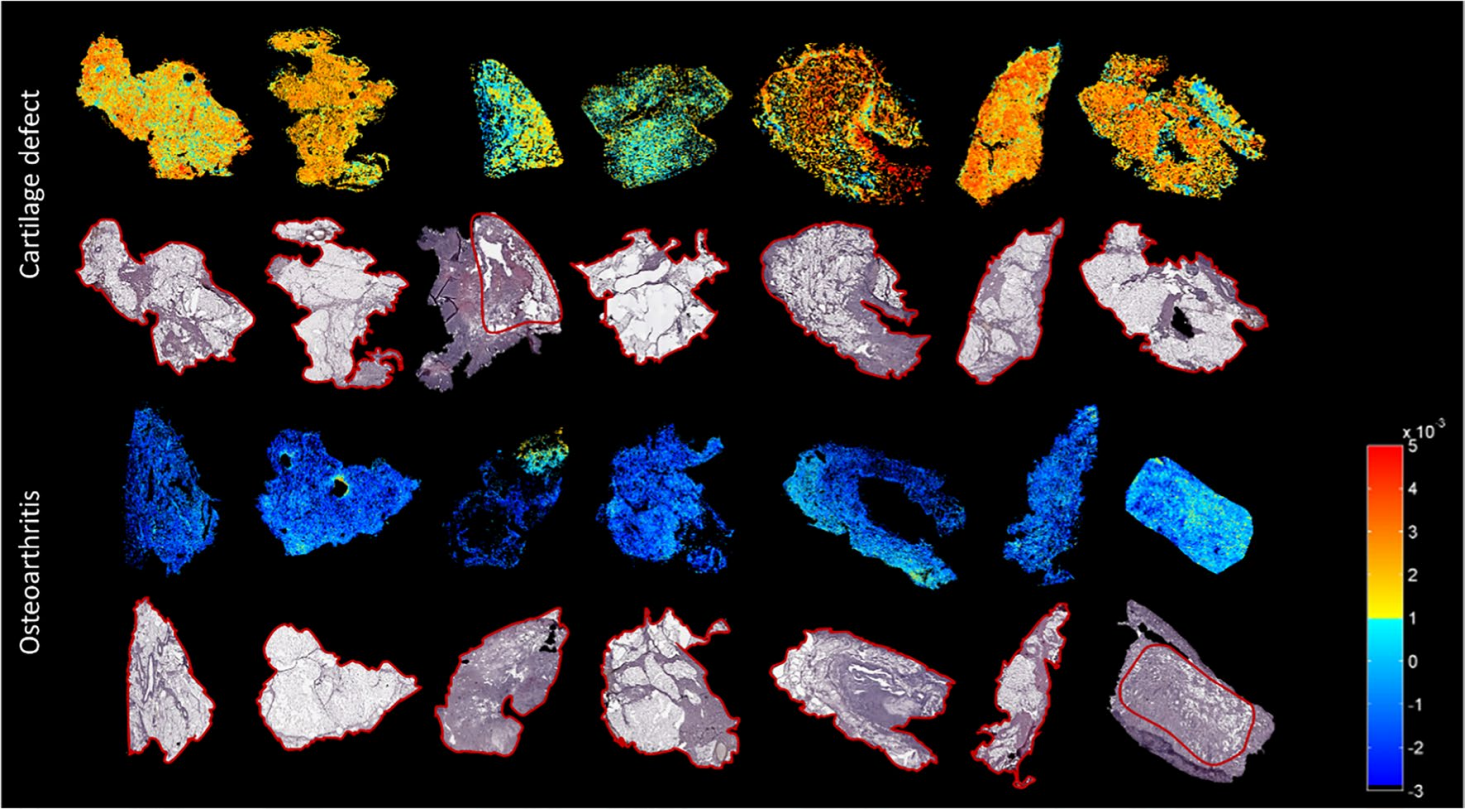
Score projection figures (discriminant function 1 (DF1)) of osteoarthritis (OA) and cartilage defect (CD) patients in negative ion mode. A separation in CD (yellow to red) and OA (blue) patient groups was visualized. The red line displays the ROI used for analysis

**Fig. 3:**
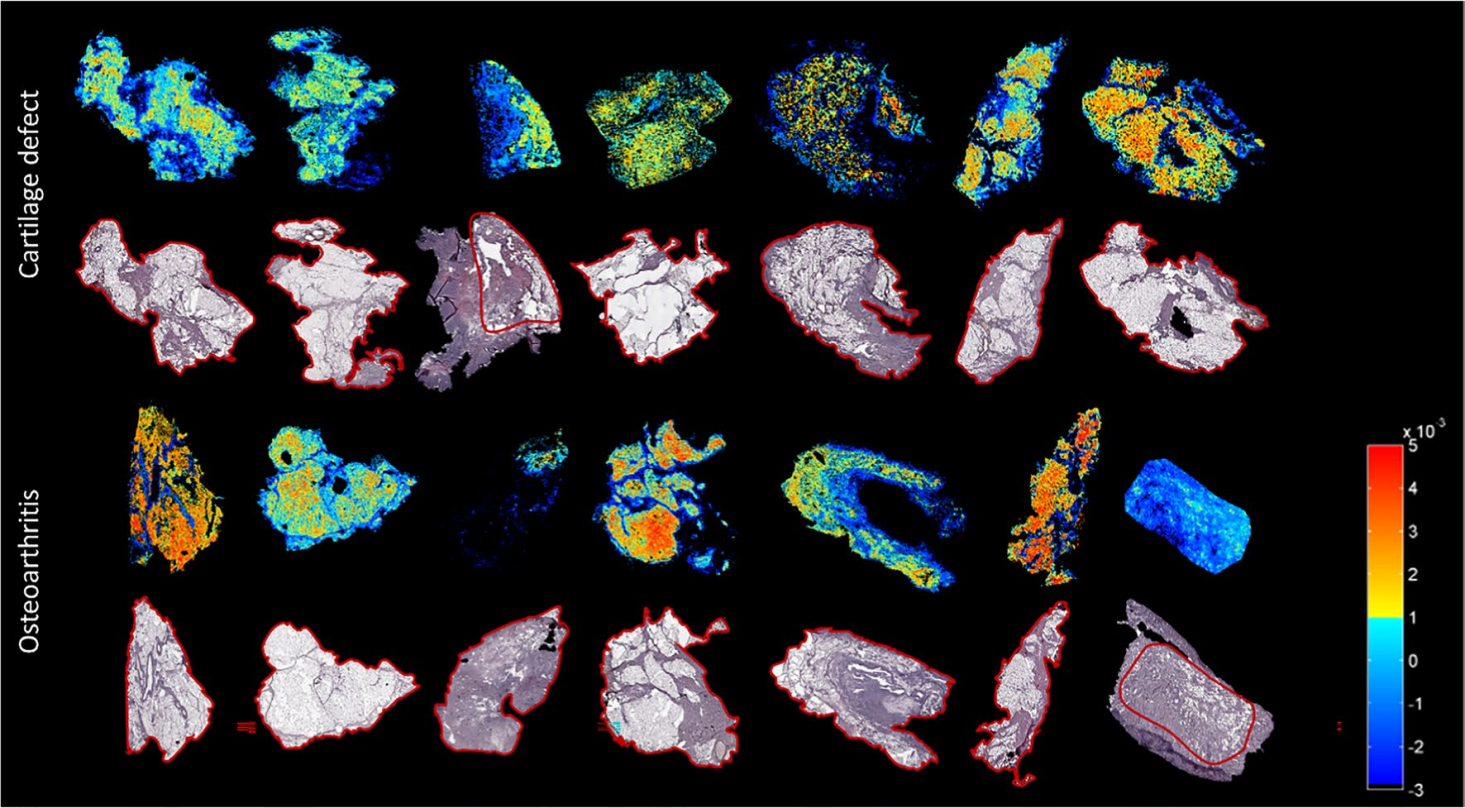
Score projection figures (discriminant function 2 (DF2)) of osteoarthritis (OA) and cartilage defect (CD) patients in negative ion mode. A separation in connective tissue (blue) and adipose tissue (yellow to red) was visualized. The red line displays the ROI used for analysis

Data analysis, after PCA and LDA, showed a separation between the group of patients with OA (*n* = 7) and the group of patients with a cartilage defect (*n* = 7) in negative ion mode. Discriminant function 1 (DF1) was differentially distributed in OA and cartilage defect patients according to the IPFP’s lipid profile, independent from tissue type (adipose tissue or connective tissue) (Fig. 1A). Likewise, in DF2, adipose tissue separated from connective tissue, independent from disease pathology (OA or cartilage defect) (Fig. 1B). The differences in DF1 can be appreciated in the score projections as well (Fig. 2), independent from tissue type (yellow to red representing cartilage defect patients and light to dark blue representing OA patients), as well as a clear separation between adipose tissue (yellow to red) and connective tissue (light to dark blue) in DF2 (Fig. 3).

In general, a higher abundance of lipids was present in the OA patient IPFP samples compared with those of cartilage defect patient IPFP samples, independently from tissue type, measured by MALDI-MSI in negative ion mode (Supplementary Table 1). In the OA patient group specifically, ether-linked phosphatidylethanolamines (PE O-38:6, PE O-38:7, PE O-36:5, and PE O-40:7) and phosphatidylethanolamines (PE 38:4 and PE 36:1) could be identified with the highest loadings (Supplementary Table 1), driving the LDA as has been shown in Fig. 1. No lipids specific for cartilage defect patients could be identified after PCA and LDA within the 20 lipids with the highest loading.

### Tissue-specific lipid profiles in negative ion mode

When comparing adipose tissue with connective tissue, PEs, PE O-s, and phosphatidylserines (PSs) were highly abundant in both tissue types. Phosphatidic acid (PA) 36:2 and phosphatidylinositol (PI) 38:3, specifically, were more abundant in adipose tissue in negative ion mode (Supplementary Table 2 and 3). More specifically, for adipose tissue, PE O-34:3, PE O-36:3, PE O-36:2, PE O-36:4, PE O-34:2, PE 34:4, PI 38:3, PA 36:2, PS 36:2, and PS 38:5 could be identified (Supplementary Table 2). For connective tissue, PE O-40:6, PE O-40:7, PE O-40:5, PE O-38:7, PE O-38:5, PE O-40:8, PE 38:4, PE 40:4, PS 36:1, and PS 40:5 were identified (Supplementary Table 3).

### Patient profiling based on IPFP lipid composition in positive ion mode

**Fig. 4:**
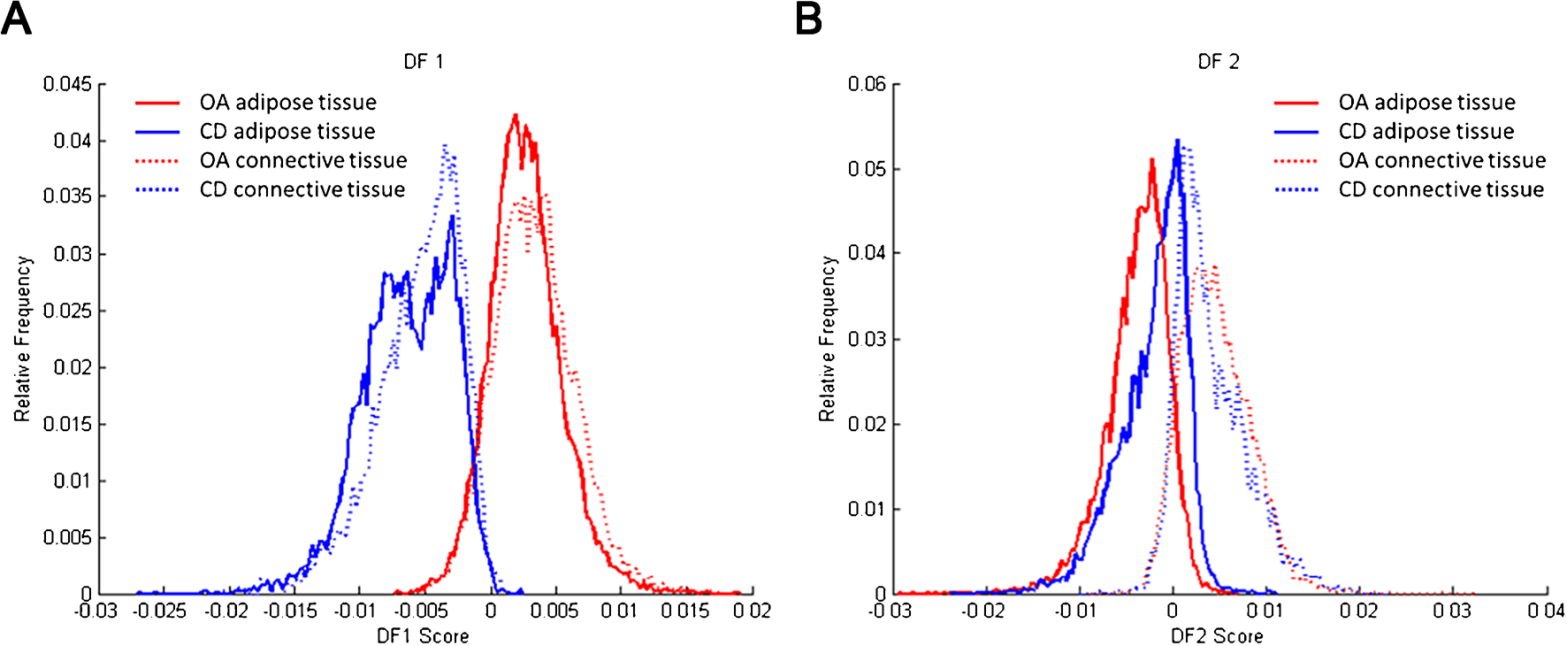
Discriminant function 1 (DF1) discriminates osteoarthritis (OA) and cartilage defect (CD) patients, independently from tissue type (adipose tissue or connective tissue) after principal component analysis (PCA) and discriminant analysis (DA) in positive ion mode (**A**, B/W = 2.0). Additionally, DF2 showed discrimination between adipose tissue (AT) of OA and CD patients and connective tissue (CT), independently from patients’ disease pathology (**B**, B/W = 1.2)

In positive ion mode, one IPFP section of the OA group and one IPFP section of the cartilage defect group were excluded from analysis because a comparison between adipose tissue and connective tissue was not possible due to the lack of or absence of either one of the tissues. Data analysis, after PCA and LDA, showed a separation between the group of patients with OA (*n* = 6) and the group of cartilage defect patients (*n* = 6) in positive ion mode. DF1 was differentially distributed between OA and cartilage defect patients according to the IPFP’s lipid profile, independent from tissue type (adipose tissue or connective tissue) (Fig. 4A). Likewise, in DF2, adipose tissue separated from connective tissue, independent from disease pathology (OA or cartilage defect) (Fig. 4B).

**Fig. 5:**
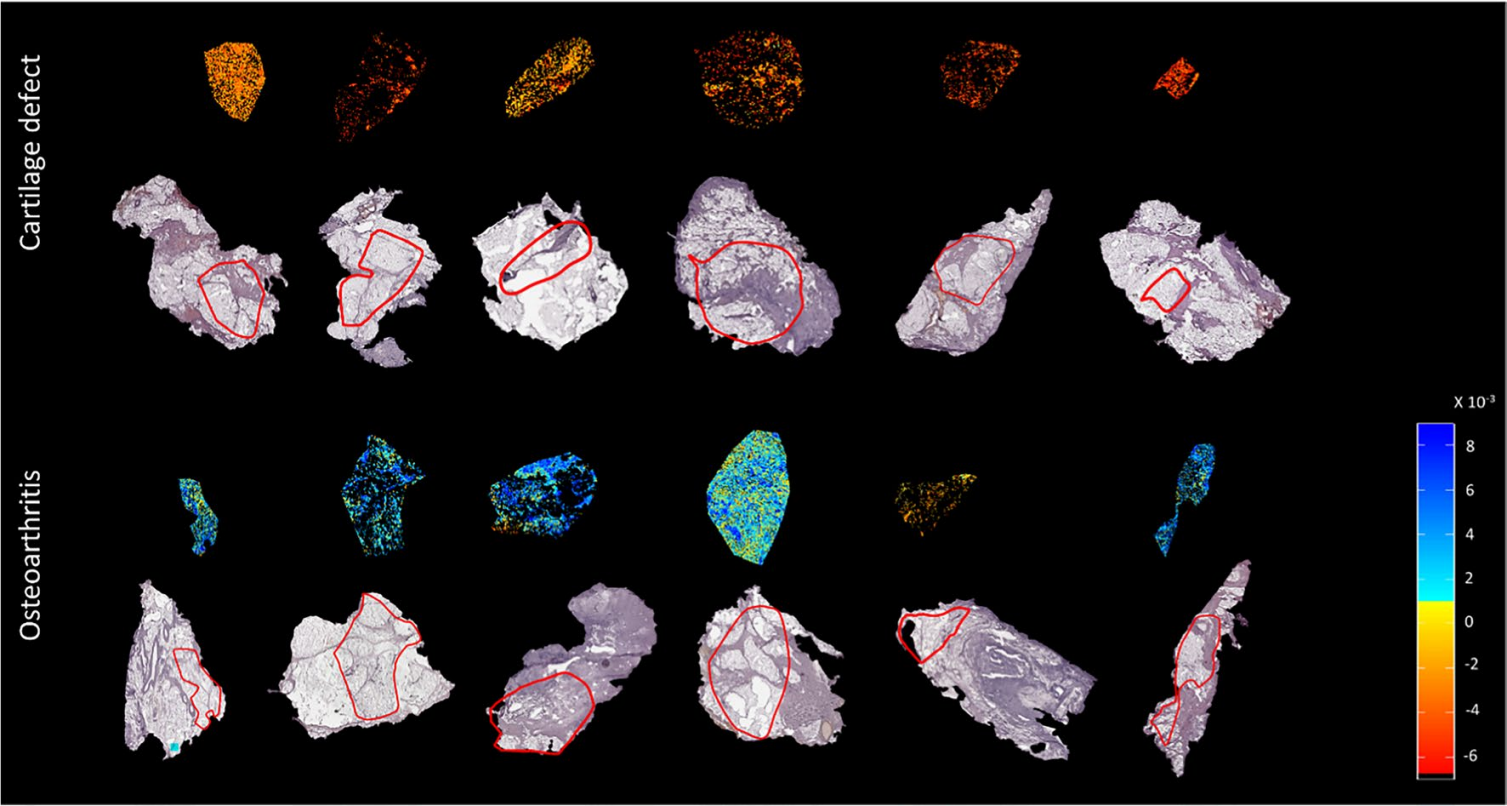
Score projection figures (discriminant function 1 (DF1)) in positive ion mode. A separation of CD (yellow to red) and OA (blue) patient groups was observed

**Fig. 6:**
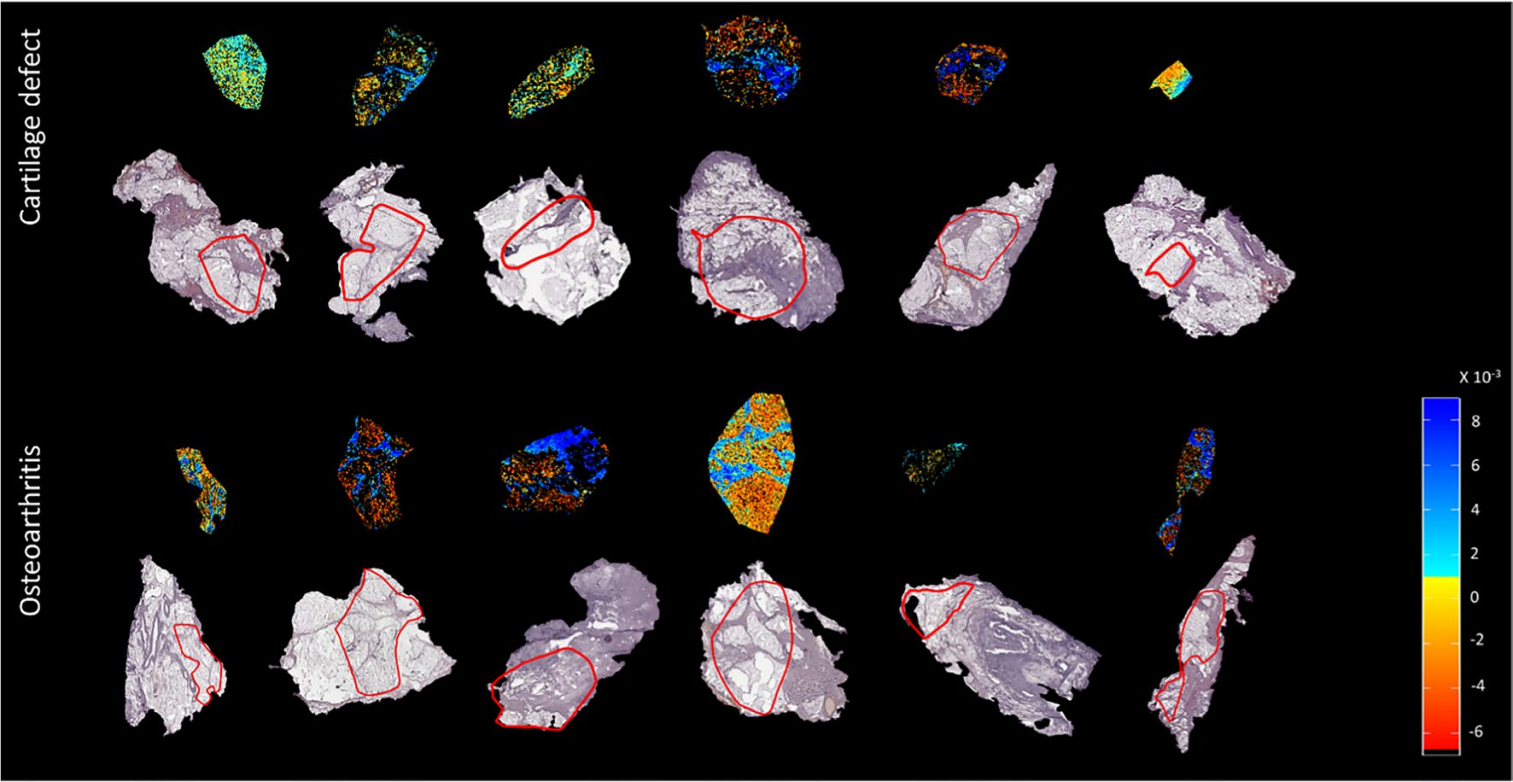
Score projection (discriminant function 2 (DF2)) in positive ion mode. A separation of connective tissue (blue) and adipose tissue (yellow to red) was observed

In addition, score projections show the same distribution of OA vs cartilage defect (Fig. 5), independent from tissue type, as well as a clear separation between adipose tissue and connective tissue (Fig. 6).

### Tissue-specific lipid profiles in positive ion mode

Whereas sphingomyelin (SM) SM 40:2;O2 and PC 36:3 were identified in the OA group in positive ion mode, only PC 36:3 could be identified for cartilage defect in the highest 20 loadings (Supplementary Table 4 and 5). PC 36:3 was thus non-specific as it was found in both OA and cartilage defect patient groups.

In positive ion mode, (ether-linked) PCs (PC O-34:2, PC O-34:3, PC O-34:3, PC O-32:2, PC O-36:3, PC 32:2, and PC 34:2) were highly abundant in the adipose tissue (Supplementary Table 6), whereas PCs (PC 38:4, PC 38:5, and PC 36:4), together with two SM (SM 42:2;O2 and SM 34:1;O2) were more abundant in the connective tissue (Supplementary Table 7).

## Discussion

This study compared for the first time the lipid profiles in the IPFP of OA and cartilage defect patients as well as specific lipids for adipose and connective tissues, using MALDI-MSI. Different lipid profiles for OA and cartilage defect patients were revealed. The IPFP phenotype of OA patients was characterized by a great plethora of phospholipids, which also has been demonstrated in the synovial fluid of early- and late-OA patients by Kosinska et al. [41].

Phospholipids contribute to the maintenance of homeostasis in a healthy joint. They function as cell membrane elements and play an important role in facilitating lubrication of the joint in synovial fluid [42–44]. Phosphocholines are essential components of the lubricating layer in the joint [44]. However, the increase of phospholipids in the synovial fluid of the OA joint results in the formation of aggregates, such as micelles [44] that disturb the lubrication of the joint. Also in line with our findings, most phospholipids show increased levels in OA synovial fluid [42, 44], contributing to OA pathogenesis by modulating inflammatory responses [41, 42].

PE O-’s, including arachidonic acid (fatty acid 20:4), were highly abundant in OA patients (Supplementary Table 1). Ether-linked lipids such as PE O- contain high amounts of arachidonic acid and have been shown to be involved in the pro-inflammatory response in diseases like obesity [45]. Eicosanoids, including prostaglandins, derived from arachidonic acid, are important inflammatory factors [46, 47] and have been shown to be involved in the inflammatory response in OA [45, 48–50]. Additionally, SM 40:2;O2 was identified as highly abundant in OA patients. Sphingolipids have been suggested as possible biomarker species in OA, as they are involved in synovial inflammation and joint repair responses [51]. An increase of SMs has been shown to be involved in the downregulation of type II collagen by disruption of the articular cartilage matrix homeostasis in vitro [52] and in bovine articular chondrocytes [53]. As an example, Kosinska et al. reported SM 42:2;O2 upregulated in patients with late OA, suggesting it as a possible biomarker for (early) OA [51].

The IPFP shows tissue heterogeneity within and between samples. Adipose tissue, connective tissue, synovial tissue, and blood vessels (dependent on tissue section) were identified using H&E histological staining (Supplementary Fig. 2). Here, we focused on the two main tissues present in the IPFP, namely adipose tissue and connective tissue. The consistency and proportion of these tissues inside the IPFP determine which lipids are detected, as well as what their importance is in disease discrimination.

The PE O-s highly present in the adipose tissue part of the IPFP did not contain arachidonic acid (Supplementary Table 2). Contrarily, the majority of the PE O-s found in the connective tissue of the IPFP did contain arachidonic acid (Supplementary Table 3), suggesting that the pro-inflammatory phenotype of OA is represented in the connective tissue of the IPFP. In general, in the tissue samples measured in this work, the IPFP of OA patients contained more connective tissue and was thus more fibrotic compared to the IPFP of cartilage repair patients. Fibrosis of especially cartilage and synovial tissue of the joint has been shown associated with OA [54, 55]. Likewise in the IPFP, fibrosis was found as a typical characteristic in OA patients, indicating that changes in the IPFP due to molecular and biomechanical alterations influence the OA development process [56, 57].

The IPFP is not commonly studied in OA research, where the focus is predominantly on synovial fluid, cartilage, or synovial membrane [19]. The importance of the IFPF in OA has already been acknowledged [23, 56–58]. However, the knowledge on the IPFP’s lipid profile and its role in OA development is limited. Because the IPFP is located between the knee joint capsule and the synovial membrane,  accessible with limited infection risk and often removed (partly or as a whole) during knee surgery, it is suggested as a promising biopsy target for diagnostic purposes and as a predictive source for OA or cartilage regenerative biomarker discovery [19]. The lipids found as specific biomarkers for OA in this research might contribute to future diagnostic applications for early OA and OA development.

By unraveling the lipidome of IPFP, we emphasize its importance, in combination with MALDI-MSI, in possible clinical diagnostic or predictive research regarding OA or cartilage regeneration. MALDI-MSI gives us a clear visualization of the lipid profile of the IPFP, taking into account the IPFP intra-tissue heterogeneity. Additional research on the lipid profile of the IPFP is necessary before a specific biomarker (profile) for OA can be identified and implementation in the clinic would be possible.

### Limitations and future research

To identify OA markers in IPFP for predictive biomarkers and possible treatment purposes, the analysis of lipid profiles in early-OA patients would be of importance. Certainly, a healthy control would be optimal, although this is not feasible due to ethical reasons. This study analyzed the lipid profile of the IPFP of end-stage OA patients, undergoing TKA. As control tissue, waste IPFP of cartilage repair surgery was analyzed. Cartilage repair patients can be considered as patients who are early in the process of OA development. Therefore, the IPFP collected from these patients is an interesting alternative. Whereas this IPFP of cartilage repair patients has been suggested to be healthier than OA IPFP, there might already be an inflammatory response present in these patients’ joints influencing the acquired results. Additionally, although patients’ IPFP were selected with care, age, BMI, gender, or the presence of synovial tissue can play a considerable role in lipid distribution. In addition, whereas MALDI-MSI gives us important information on the spatial distribution of molecules throughout the tissue, differences in ionization or signal intensity per pixel prevent us from measuring the amount of the analyte of interest present. Validation of our results is necessary and can possibly be done by cutting out the region of interest (e.g., connective tissue) within the IPFP using laser microdissection (LMD) prior to quantitative liquid chromatography tandem MS (LC–MS/MS) [59].

The IPFP is suggested as a potential source of biomarkers for OA. Compared to more favorably explored fluids or tissues such as synovial fluid or cartilage, the IPFP is (almost) always present in the affected knee, easy to biopsy with limited infection risk, and commonly removed as waste material during cartilage repair surgery or TKA, thus highly available for research purposes. It is suggested that future experiments should be performed on larger patient groups and that not only inter-patient variability, but also intra-tissue heterogeneity, should be taken into account. Possible biomarkers within the IPFP might relate to the genesis of OA and lead to possible novel interventions, treatments, or drug targets.

## Conclusion

In conclusion, several lipid species were identified that are characteristic of OA and cartilage defect phenotypes. Highly expressed PE O-s, containing arachidonic acid, in the connective tissue of the IPFP, suggested a connection of these lipids to the OA phenotype. Intra-tissue heterogeneity has been shown of importance and might be associated with the different phenotypes.

### Supplementary Information

Below is the link to the electronic supplementary material.

Supplementary file1 (DOCX 2482 KB)

## Abbreviations

BMI: Body mass index
B/W: Between/within
CID: Collision-induced dissociation
DDA: Data-dependent acquisition
DF: Discriminant function
H&E: Hematoxylin and eosin
HCD: Higher-energy collisional dissociation
IPFP: Infrapatellar fat pad
ITO: Indium tin oxide
KL: Kellgren-Lawrence
LC–MS/MS: Liquid chromatography tandem mass spectrometry
LDA: Linear discriminant analysis
LMD: Laser microdissection
MALDI-MSI: Matrix-assisted laser desorption/ionization mass spectrometry imaging
MEC: Medical ethics committee
MRI: Magnetic resonance imaging
MS: Mass spectrometry
MUMC +: Maastricht University Medical Center +
NCE: Normalized collision energy
*m/z*: Mass-to-charge ratio
OA: Osteoarthritis
PA: Phosphatidic acid
PBS: Phosphate-buffered saline
PCA: Principal component analysis
PE: Phosphatidylethanolamine
PE O-: Ether-linked phosphatidylethanolamine
PI: Phosphatidylinositol
PS: Phosphatidylserine
ROI: Region of interest
SM: Sphingomyelin
T2DM: Type 2 diabetes mellitus
TKA: Total knee arthroplasty
WMO: Wet medisch-wetenschappelijk onderzoek

## Declarations

### Source of biological material

Human infrapatellar fat pads were collected as surgical waste material from patients with moderate- to late-stage OA undergoing total knee arthroplasty (TKA) and control patients undergoing cartilage repair surgery for an osteochondral defect in a femoral condyle at the Maastricht University Medical Center + (MUMC +).
