# Supplementary material for: Matrix-assisted laser desorption/ionization mass spectrometry imaging (MALDI-MSI) reveals potential lipid markers between infrapatellar fat pad biopsies of osteoarthritis and cartilage defect patients
Source: Anal Bioanal Chem. 2023 Jul 28;415(24):5997–6007. doi: 10.1007/s00216-023-04871-9 (PMC10556153; doi:10.1007/s00216-023-04871-9)
Supplement: Supplementary file 1 — Supplementary file1 (DOCX 2482 KB) [file 216_2023_4871_MOESM1_ESM.docx]

**Supplementary material**


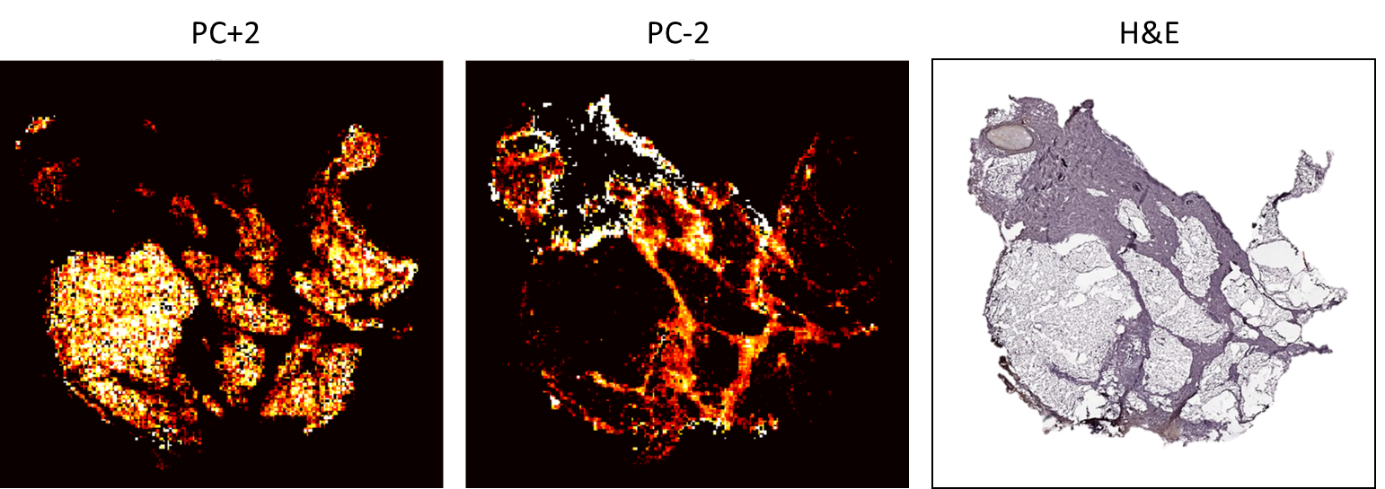
**Supplementary Figure 1:** An example in which adipose tissue (PC+2) and connective tissue (PC-2) could be separated using principal component analysis (PCA) and linear discriminant analysis (LDA), showing the distribution of lipids based in negative ion mode.


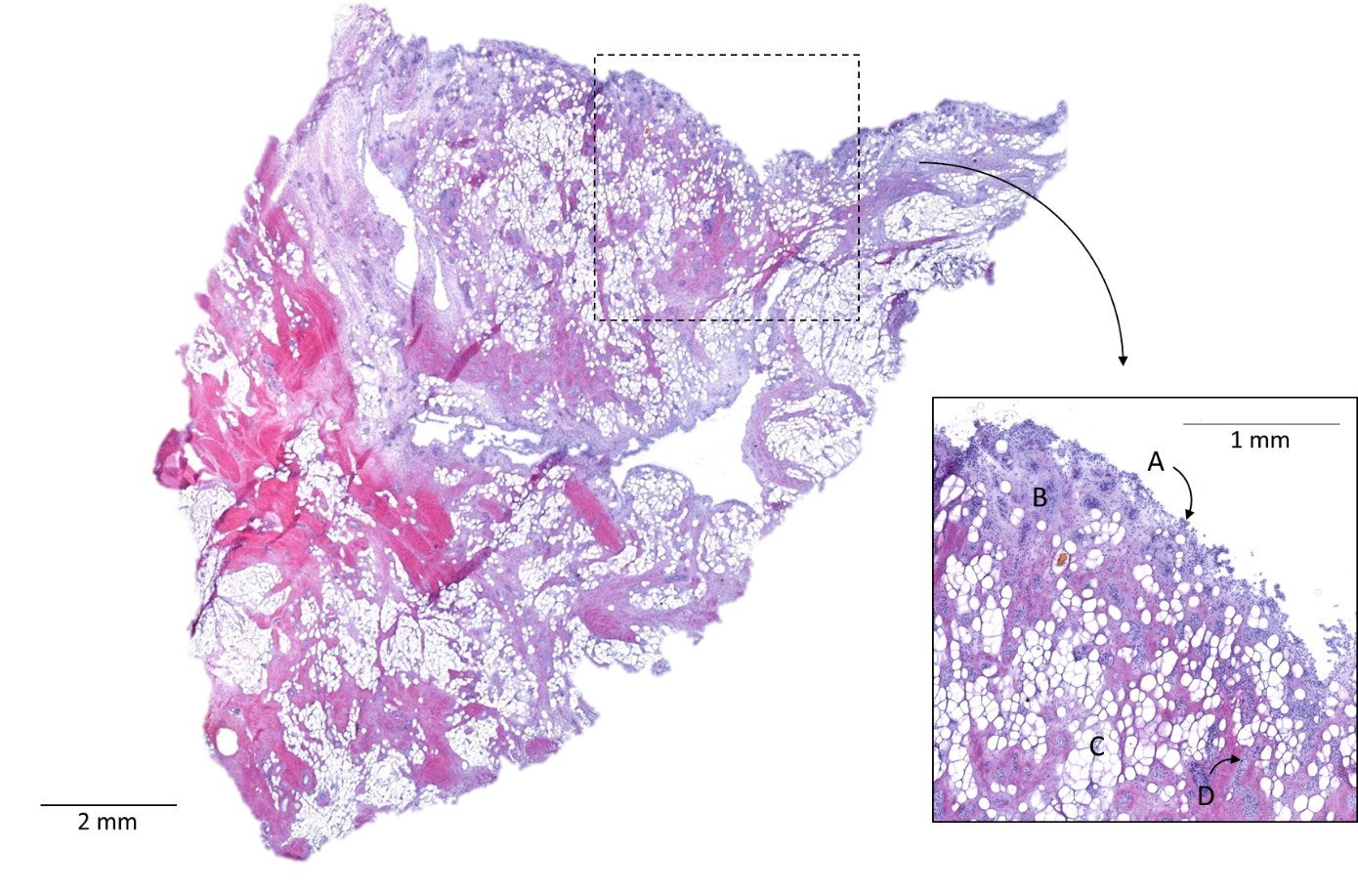


**Supplementary Figure 2:** Example of a Hematoxylin and Eosin stained infrapatellar fat pad tissue section. The synovial membrane (A), connective tissue (B), adipose tissue (C) and blood vessels (D) could be identified.

**Supplementary Table 1:** Lipid assignments for osteoarthritis patients (DF1) based on MS^2^ data in negative ion mode. The *m/z* values were collected on an Orbitrap Elite running in data-dependent acquisition mode.

| **Type** | **Ion mode** | **Rapiflex MS^1^ (m/z)** | **Loading** | **Orbitrap MS^1^ (m/z)** | **Δ ppm** | **Lipid ID** | **Adduct** |
| --- | --- | --- | --- | --- | --- | --- | --- |
| OA | Negative | 748.6 | -0.351 | 748.5286 | 0.1 | PE O-38:6  PE O-18:2_20:4  PE O-16:1_22:5 | [M-H]^-^ |
| OA | Negative | 766.6 | -0.351 | 766.5391 | 0.1 | PE 38:4  PE 18:0_20:4 | [M-H]^-^ |
| OA | Negative | 746.6 | -0.343 | 746.5130 | 0.0 | PE O-38:7  PE O-16:1_22:6  PE O-18:3_20:4 | [M-H]^-^ |
| OA | Negative | 722.6 | -0.328 | 722.5130 | 0.0 | PE O-36:5  PE O-16:1_20:4 | [M-H]^-^ |
| OA | Negative | 744.6 | -0.314 | 744.5548 | 0.1 | PE 36:1  PE 18:0_18:1  PE 16:0_20:1 | [M-H]^-^ |
| OA | Negative | 774.6 | -0.291 | 774.5442 | 0.1 | PE O-40:7  PE O-18:1_22:6  PE O-22:6_18:1  PE O-18:2_22:5  PE O-20:3_20:4 | [M-H]^-^ |

*m/z* = mass to charge ratio | OA = osteoarthritis | PE = phosphatidylethanolamine | PE O = ether-linked phosphatidylethanolamine

**Supplementary Table 2:** Lipid assignments for adipose tissue in osteoarthritis and cartilage defect patients (DF2) based on MS^2^ data in negative ion mode. The *m/z* values were collected on an Orbitrap Elite running in data-dependent acquisition mode.

| **Type** | **Ion mode** | **Rapiflex MS^1^ (m/z)** | **Loading** | **Orbitrap MS^1^ (m/z)** | **Δ ppm** | **Lipid ID** | **Adduct** |
| --- | --- | --- | --- | --- | --- | --- | --- |
| AT | Negative | 698.6 | 0.662 | 698.5130 | 0.0 | PE O-34:3  PE O-16:1_18:2  PE O-18:2_16:1 | [M-H]^-^ |
| AT | Negative | 726.6 | 0.648 | 726.5443 | 0.0 | PE O-36:3  PE O-18:1_18:2  PE O-18:2_18:1 | [M-H]^-^ |
| AT | Negative | 699.6 | 0.636 | 699.4971 | 0.1 | PA 36:2  PA 18:0_18:2  PA 18:1_18:1 | [M-H]^-^ |
| AT | Negative | 786.6 | 0.568 | 786.5290 | 0.1 | PS 36:2  PS 18:0_18:2  PS 18:1_18:1 | [M-H]^-^ |
| AT | Negative | 728.6 | 0.553 | 728.5599 | 0.1 | PE O-36:2  PE O-18:1_18:1  PE O-18:0_18:2 | [M-H]^-^ |
| AT | Negative | 724.6 | 0.547 | 724.5286 | 0.1 | PE O-36:4  PE O-18:2_18:2  PE O-16:1_20:3 | [M-H]^-^ |
| AT | Negative | 808.6 | 0.459 | 808.5112 | 2.7 | PS 38:5  PS 18:0_20:5  PS 18:1_20:4  PS 16:1_22:4  PS 18:2_20:3 | [M-H]^-^ |
| AT | Negative | 700.6 | 0.443 | 700.5287 | 0.1 | PE O-34:2  PE O-16:1_18:1  PE O-16:0_18:2  PE O-18:1_16:1  PE O-18:2_16:0 | [M-H]^-^ |
| AT | Negative | 883.6 | 0.428 | 883.5341 | 0.1 | PI 38:3  PI 38:1_20:4 | [M-H]^-^ |
| AT | Negative | 714.6 | 0.399 | 714.5080 | 0.1 | PE 34:2  PE 16:0_18:2  PE 16:1_18:1 | [M-H]^-^ |

AT = adipose tissue | *m/z* = mass to charge ratio | PA = phosphatidic acid | PE = phosphatidylethanolamine | PE O = ether-linked phosphatidylethanolamine | PI = phosphatidylinositol | PS = phosphatidylserine

**Supplementary Table 3:** Lipid assignments for connective tissue in osteoarthritis and cartilage defect patients (DF2) based on MS^2^ data in negative ion mode. The *m/z* values were collected on an Orbitrap Elite running in data-dependent acquisition mode.

| **Type** | **Ion-mode** | **Rapiflex MS^1^ (m/z)** | **Loading** | **Orbitrap MS^1^ (m/z)** | **Δ ppm** | **Lipid ID** | **Adduct** |
| --- | --- | --- | --- | --- | --- | --- | --- |
| CT | Negative | 810.6 | 0.459 | 810.5279 | 1.4 | PS 38:4  PS 18:0_20:4  PS 18:1_20:3 | [M-H]^-^ |
| CT | Negative | 776.6 | 0.439 | 776.5598 | 0.2 | PE O-40:6  PE O-20:2_20:4  PE O-18:2_22:4  PE O-18:1_22:5 | [M-H]^-^ |
| CT | Negative | 774.6 | 0.427 | 774.5442 | 0.1 | PE O-40:7  PE O-18:1_22:6  PE O-22:6_18:1  PE O-18:2_22:5  PE O-20:3_20:4 | [M-H]^-^ |
| CT | Negative | 778.6 | 0.426 | 778.5754 | 0.2 | PE O-40:5  PE O-20:2_20:4  PE O-18:2_22:4  PE O-18:1_22:5 | [M-H]^-^ |
| CT | Negative | 766.6 | 0.403 | 766.5391 | 0.1 | PE 38:4  PE 18:0_20:4 | [M-H]^-^ |
| CT | Negative | 746.6 | 0.399 | 746.5130 | 0.0 | PE O-38:7  PE O-16:1_22:6  PE O-18:3_20:4 | [M-H]^-^ |
| CT | Negative | 750.6 | 0.386 | 750.5442 | 0.1 | PE O-38:5  PE O-18:1_20:4  PE O-16:1_22:4 | [M-H]^-^ |
| CT | Negative | 794.6 | 0.385 | 794.5703 | 0.2 | PE 40:4  PE 20:0_20:4  PE 18:0_22:4 | [M-H]^-^ |
| CT | Negative | 788.6 | 0.383 | 788.5446 | 0.1 | PS 36:1  PS 18:0_18:1 | [M-H]^-^ |
| CT | Negative | 836.6 | 0.347 | 836.5445 | 0.3 | PS 40:5  PS 18:0_22:5 | [M-H]^-^ |
| CT | Negative | 772.6 | 0.342 | 772.5286 | 0.2 | PE O-40:8  PE O-18:2_22:6 | [M-H]^-^ |

CT = connective tissue | *m/z* = mass to charge ratio | MMPE = monomethyl-phosphatidylethanolamine | PE = phosphatidylethanolamine | PE O = ether-linked phosphatidylethanolamine | PG = phosphatidylglycerol | PS = phosphatidylserine

**Supplementary Table 4:** Lipid assignments for osteoarthritis patients (DF1) based on MS^2^ data in positive ion mode. The *m/z* values were collected on an Orbitrap Elite running in data dependent acquisition mode.

| **Type** | **Ion-mode** | **Rapiflex MS^1^ (m/z)** | **Loading** | **Orbitrap MS^1^ (m/z)** | **Δ ppm** | **Lipid ID** | **Adduct** |
| --- | --- | --- | --- | --- | --- | --- | --- |
| OA | Positive | 785.7 | 0.169 | 785.6529 | 0.2 | SM 40:2;O2 | [M+H]^+^ |
| OA | Positive | 806.7 | 0.178 | 806.5670 | 0.0 | PC 36:3 | [M+Na]^+^ |
| OA | Positive | 784.7 | 0.163 | 784.5847 | 0.5 | PC 36:3 | [M+H]^+^ |

OA = osteoarthritis | *m/z* = mass to charge ratio | PC = phosphatidylcholine | SM = sphingomyelin

**Supplementary Table 5:** Lipid assignments for cartilage defect patients (DF1) based on MS^2^ data in positive ion mode. The *m/z* values were collected on an Orbitrap Elite running in data dependent acquisition mode.

| **Type** | **Ion-mode** | **Rapiflex MS^1^ (m/z)** | **Loading** | **Orbitrap MS^1^ (m/z)** | **Δ ppm** | **Lipid ID** | **Adduct** |
| --- | --- | --- | --- | --- | --- | --- | --- |
| CD | Positive | 806.5 | -0.253 | 806.5670 | 0.0 | PC 36:3 | [M+Na]^+^ |

CD = cartilage defect | *m/z* = mass to charge ratio | PC = phosphatidylcholine

**Supplementary Table 6:** Lipid assignments for adipose tissue in osteoarthritis and cartilage defect patients (DF2) based on MS^2^ data in positive ion mode. The *m/z* values were collected on an Orbitrap Elite running in data dependent acquisition mode.

| **Type** | **Ion-mode** | **Rapiflex MS^1^ (m/z)** | **Loading** | **Orbitrap MS^1^ (m/z)** | **Δ ppm** | **Lipid ID** | **Adduct** |
| --- | --- | --- | --- | --- | --- | --- | --- |
| AT | Positive | 744.5 | 0.566 | 744.5900 | 0.2 | PC O-34:2 | [M+H]^+^ |
| AT | Positive | 742.7 | 0.472 | 742.5744 | 0.2 | PC O-34:3 | [M+H]^+^ |
| AT | Positive | 764.7 | 0.419 | 764.5563 | 0.1 | PC O-34:3 | [M+Na]^+^ |
| AT | Positive | 730.6 | 0.416 | 730.5380 | 0.2 | PC 32:2 | [M+H]^+^ |
| AT | Positive | 758.5 | 0.393 | 758.5690 | 0.3 | PC 34:2 | [M+H]^+^ |
| AT | Positive | 716.6 | 0.373 | 716.5588 | 0.1 | PC O-32:2 | [M+H]^+^ |
| AT | Positive | 770.7 | 0.372 | 770.6055 | 0.4 | PC O-36:3 | [M+H]^+^ |

AT = adipose tissue | *m/z* = mass to charge ratio | PC = phosphatidylcholine | PC O- = ether-linked phosphatidylcholine

**Supplementary Table 7:** Lipid assignments for connective tissue in osteoarthritis and cartilage defect patients (DF2) based on MS^2^ data in positive ion mode. The *m/z* values were collected on an Orbitrap Elite running in data dependent acquisition mode.

| **Type** | **Ion-mode** | **Rapiflex MS^1^ (m/z)** | **Loading** | **Orbitrap MS^1^ (m/z)** | **Δ ppm** | **Lipid ID** | **Adduct** |
| --- | --- | --- | --- | --- | --- | --- | --- |
| CT | Positive | 832.7 | -0.317 | 832.5827 | 0.0 | PC 38:4 | [M+Na]^+^ |
| CT | Positive | 830.7 | -0.304 | 830.5670 | 0.0 | PC 38:5 | [M+Na]^+^ |
| CT | Positive | 813.8 | -0.292 | 813.6843 | 0.2 | SM 42:2;O2 | [M+H]^+^ |
| CT | Positive | 725.7 | -0.291 | 725.5567 | 0.1 | SM 34:1;O2 | [M+Na]^+^ |
| CT | Positive | 804.7 | -0.279 | 804.5513 | 0.1 | PC 36:4 | [M+Na]^+^ |

*m/z* = mass to charge ratio | PA = phosphatidic acid | PC = phosphatidylcholine | SM = sphingomyelin
